# A ResNet attention model for classifying mosquitoes from wing-beating sounds

**DOI:** 10.1038/s41598-022-14372-x

**Published:** 2022-06-20

**Authors:** Xutong Wei, Md Zakir Hossain, Khandaker Asif Ahmed

**Affiliations:** 1 Research School of Computing, Australian National University, Canberra, ACT 2601 Australia; 2 Biological Data Science Institute, Australian National University, Canberra, ACT 2601 Australia; 3 CSIRO Agriculutre and Food, Black Mountain, Canberra, ACT 2601 Australia; 4 CSIRO Land and Water, Black Mountain, Canberra, ACT 2601 Australia

**Keywords:** Classification and taxonomy, Image processing, Machine learning

## Abstract

Mosquitoes are vectors of numerous deadly diseases, and mosquito classification task is vital for their control programs. To ease manual labor and time-consuming classification tasks, numerous image-based machine-learning (ML) models have been developed to classify different mosquito species. Mosquito wing-beating sounds can serve as a unique classifier for mosquito classification tasks, which can be adopted easily in field applications. The current study aims to develop a deep neural network model to identify six mosquito species of three different genera, based on their wing-beating sounds. While existing models focused on raw audios, we developed a comprehensive pre-processing step to convert raw audios into more informative Mel-spectrograms, resulting in more robust and noise-free extracted features. Our model, namely *’Wing-beating Network’ or ’WbNet’*, combines the state-of-art residual neural network (ResNet) model as a baseline, with self-attention mechanism and data-augmentation technique, and outperformed other existing models. The *WbNet* achieved the highest performance of 89.9% and 98.9% for WINGBEATS and ABUZZ data respectively. For species of *Aedes* and *Culex* genera, our model achieved 100% precision, recall and F1-scores, whereas, for *Anopheles*, the *WbNet* reached above 95%. We also compared two existing wing-beating datasets, namely WINGBEATS and ABUZZ, and found our model does not need sophisticated audio devices, hence performed better on ABUZZ audios, captured on usual mobile devices. Overall, our model has potential to serve in mosquito monitoring and prevalence studies in mosquito eradication programs, along with potential implementation in classification tasks of insect pests or other sound-based classifications.

## Introduction

Machine learning (ML) models are being implemented widely in automatic classification tasks^1^. ML models are capable of extracting and processing classification features by ensuring time-efficiency and minimal human intervention^2^. Besides their wide application in diverse fields, they are being applied in numerous insect classifications tasks. An image-based Convolutional Neural Network (CNN) model^3^ identified different insect pests in agricultural crops to improve a healthy food supply. Valan et al.^4^ used a CNN model, pre-trained on the general dataset (imageNet), and transferred the trained features for the insect classification task. Their high performing model showed the potentiality for transfer learning^5^ techniques in classification projects, reducing the need of creating a large training dataset.

Moreover, numerous traditional ML methods such as Support Vector Machine (SVM)^6^, Naive Bayes^7^, and K-Nearest Neighbours (KNN)^8^ have been adopted to classify different insect species. Image-based ML models have been widely used in mosquito systematics. The venation and shape of mosquito wings are species-specific, and Artificial Neural Network (ANN) classification models on mosquito wing images showed good accuracies in mosquito species classification^9,10^. However, the collection of wing images is a cumbersome task, which required long and sophisticated mounting and image capture procedures to get a single informative image^11^. Rather, several CNN-based models have been developed to extract features and classify different mosquito species based on the whole body and posture images^11–13^. Most of the studies utilised manually curated datasets, with similar backgrounds—which is a cumbersome task for creating a large dataset. Recently, Yefeng et al.^14^ developed a ML-based approach which utilises open-sourced insect images, to filter in informative fruitfly images, regardless of diverse backgrounds and showed the potential of the dataset for fruitfly classification tasks. However, image-based insect classification favor larger size insects, where it is relatively easier to extract visual features. For small insects, it often becomes difficult to capture good quality images, and sometimes complex backgrounds make the detection task more challenging. Besides image-based classification, several ML models are being utilised for different classifiers on audios, odorant^15^ or molecular datasets^16,17^.

Insects produce a wide range of sounds, ranging from their eating, moving, wing-beating during flight, and these sounds can be used as unique classifiers to classify specific insect classes. Fine Gaussian SVM and KNN algorithms build on numerous insect sounds^18^ are able to classify some insects classes, whereas, another Bayesian model for insect flight sounds^19^ showed improved performance for insect classification tasks. Besides, some ANN models such as Probabilistic Neural Network^20^ and deep learning algorithms such as CNNs^21^, were also been used in insect sounds classification and detection. Some algorithms used raw audios as input^22^, whereas other models did rigorous pre-processing tasks, including Mel-frequency Cepstral Coefficients (MFCCs) features of audio waveforms^20^ to make their models more robust.

Mosquitoes produce unique and species-specific wing-beating sounds^23^. Numerous studies utilized publicly available mosquito wing-beating datasets, namely WINGBEATS^24^ and ABUZZ^25^ to classify different mosquitoes. A CNN model on ABUZZ dataset^25^ showed 97.65% accuracy for binary classification of *Aedes aegypti*, but for multi-class classification of twenty mosquito species, the average accuracy dropped to 78.12%^26^. A DenseNet-121 based CNN model^27^ on WINGBEATS^24^ dataset achieved 96% accuracy to classify six mosquito species. The model extracted audio features and trained on six mosquito species from spectrograms. Moreover, a 1D CNN model with a combination of Long Short-Term Memory (LSTM) network^28^ can feed the raw wing beating audios directly into the network without any preprocessing procedures. However, raw audio signals contain only time-domain information, and it is often difficult to obtain information about frequency distribution. Spectrograms have advantage over raw audio signals by considering the frequency distribution changes over time from two dimensions, which allows ML models to extract more features for the classification task. A Mel-spectrogram is a type of spectrogram where applied mel-scale in the frequency domain in a spectrogram^29^. Compared with the ordinary linear spectrogram, Mel-spectrogram is closer to the sound frequency recognition of the human ear, and the difference in the discrimination of low-frequency sounds is greater. Mel-spectrogram is the result of some non-linear transformation of the frequency scale which shows in Eq. (1), where *f* is the frequency.The mosquitoes’ wing beating frequency ranges between 100 and 1000 Hz^30^, which belongs to low-frequency sounds, so, transforming mosquito wing-beating audio sounds to Mel-spectrograms can obtain more useful information, so as to potentially perform better for classification tasks.

1 $$\begin{aligned} mel(f) = 2595 \times \log \left( 1 + \frac{f}{700}\right) \end{aligned}$$

Wing beating sounds and relevant ML models have implications in mosquito systematics and potentiality in public health. Mosquitoes are the vector of numerous deadly pathogens, resulting in yellow fever, encephalitis viruses, malaria, West Nile virus, chikungunya, Rift Vally fever, dengue^31,32^. Besides, according to World Health Organization, every year millions of people get infected with mosquito-borne diseases worldwide and over 400,000 deaths per year are resulted from the Malaria alone^33^. Specific mosquito genera and species spread specific types of diseases and before any species-specific control programs, it is necessary to classify mosquito species efficiently and robustly. The classification task will also be helpful to measure population density within a particular area and take necessary initiatives for an eco-friendly and sustainable mosquito control strategy. Generally, mosquitoes are classified based on their morphological features^34^ and individual sexes of each mosquito species show differences in their antennal features^35^. It is often cumbersome to distinguish mosquitoes only by tiny morphological features, and molecular identification methods such as mitochondrial DNA-based barcode technology^36,37^ become more effective way to distinguish different mosquito species. Both of the methods are expensive and time-consuming, need particular domain experts to perform the tasks. There is a need for ML models to identify suitable classifiers to classify mosquito species and also a robust model for detection.

The current study aims to build a Deep Neural Network based-classification model to classify different mosquito species. Wing beating sounds of six mosquito species of three different genera, namely *Aedes*, *Anopheles*, and *Culex* were collected from two different publicly available datasets, rigorously pre-processed, transformed into Mel-spectrograms. Later an augmentation method was applied, and tested on different architectures for species classification. Finally, a ResNet-based model *WbNet* was developed, with a combination of the self-attention mechanism. Our robust model has implications in mosquito systematics tasks, and can be extended further for gender-based classification. The model can be deployed easily in different remote areas to monitor prevalence of specific mosquito species and will be helpful to prevent mosquito-borne diseases by developing species-specific control measures.

## Results

**Figure 1:**
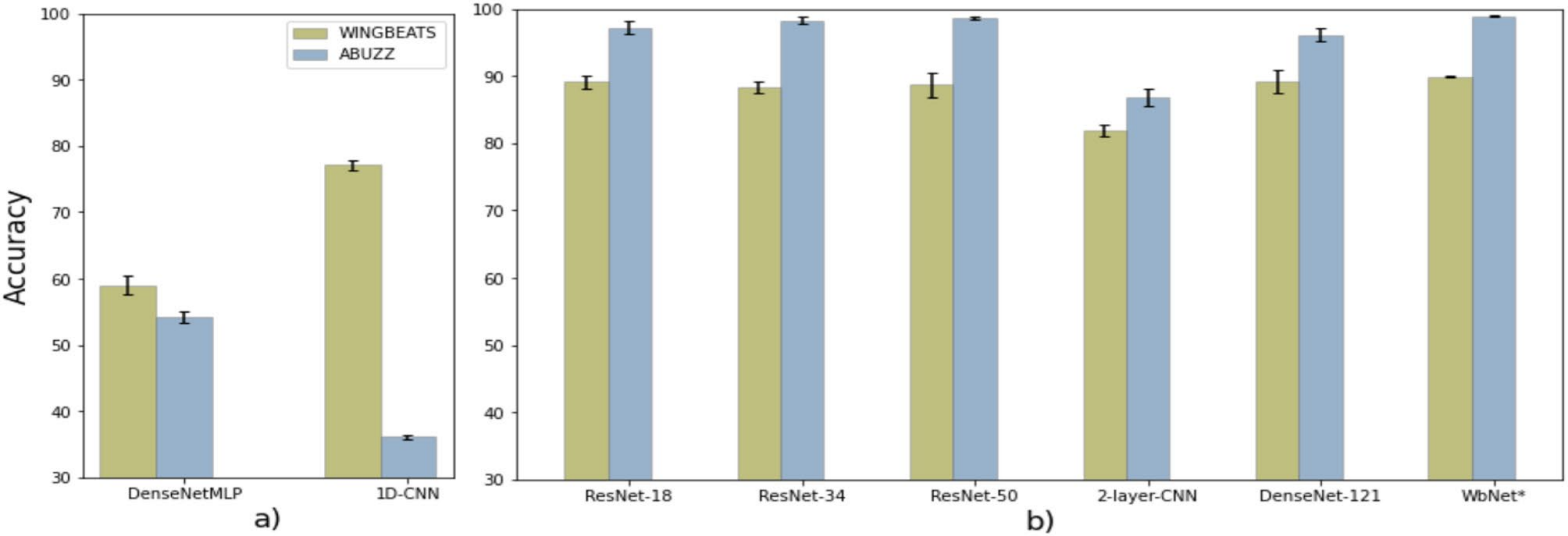
Models comparison with input: (**a**) raw audio waveforms, (**b**) pre-processed spectrograms.

From the spectrogram-based models, we found that our model, namely *WbNet*, has outperformed other ML models (Fig. 1). In Fig. 1, it is shown that except *WbNet*, ResNet-18 got the best accuracy of 89.1% for WINGBEATS, whereas, ResNet-34 performed best for ABUZZ (98.3%). We also found that all models consistently well-performed on the ABUZZ dataset compared to the WINGBEATS dataset. For example, ResNet-18 got 89.1% and 97.2% accuracies in WINGBEATS and ABUZZ. Moreover, we compared the ResNet models with two existing models (2-layer-CNN and DenseNet-121) on mosquito wing beating sounds. A multi-class classifier built on a 2-layer convolutional neural network^26^ got 81.9% and 86.9% accuracies on WINGBEATS and ABUZZ. Another DenseNet-121 based CNN model^27^ with our pre-processed data, got accuracies of 89.2% and 96.1% on WINGBEATS and ABUZZ.

WbNet had been developed and implemented as a new ResNet-Attention model, for mosquito species classification problem. The *WbNet* model (shown in Fig. 4) is built upon ResNet-18 network, with residual blocks to ensure efficient learning. A self-attention mechanism was also added within the model to persuasively capture the global spatial dependencies and solve the forgotten phenomenon of data—which might exist in relatively long audios. In addition, rigorous data pre-processing and data augmentation techniques had also been applied before feeding the data into our model. Our model achieved accuracies of $89.9\pm 0.15\%$ and $98\pm 0.09\%$ for WINGBEATS and ABUZZ respectively (Fig. 1). A detailed analysis based on each mosquito species is illustrated in Table 1. It is worthwhile to note that we did not report any accuracy in Table 1 as the Table illustrates the individual performances for each species, i.e. locally, where accuracy and precision are used to report performances globally and locally respectively. Due to the nature of the evaluation matrix, precision is good fit for measuring individual performance for each mosquito species (accuracy measures general performance across all species).

**Table 1:** *WbNet* evaluation metrics for different mosquito species.

Species	WINGBEATS dataset			ABUZZ dataset		
	Precision	Recall	F1-score	Precision	Recall	F1-score
*Ae. aegypti*	91	90	91	100	100	100
*Ae. albopictus*	91	99	95	100	100	100
*An. arabiensis*	67	69	68	100	95	97
*An. gambiae*	90	89	89	97	100	98
*Cu. pipiens*	96	93	94	100	100	100
*Cu. quinquefasciatus*	87	92	89	100	100	100

**Figure 2:**
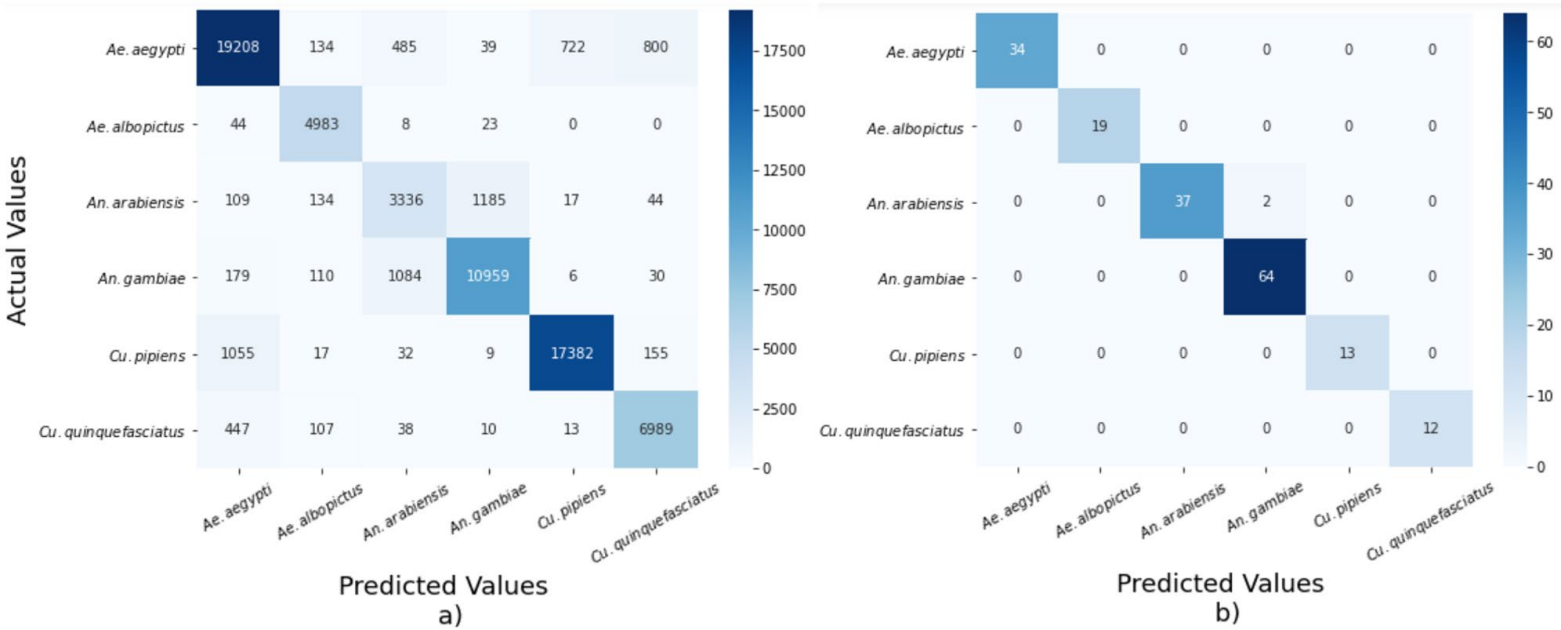
Confusion matrix of *WbNet* on (**a**) WINGBEATS, (**b**) ABUZZ.

Overall, we found that, *Ae. albopictus* species has the best classification scores in both datasets by showing 100% precision, recall and F1-score for ABUZZ, and 91% Precision, 99% Recall, 95% F1-score for WINGBEATS. Even though there are marked discrepancies among different species classification scores, we showed that our classification model works well for ABUZZ data with near-perfect accuracy. In the WINGBEATS dataset, *An. arabiensis* got the lowest precision (67%), recall (69%), and F1-score (68%), whereas, *Cu. pipiens* has reached the highest precision of 96%, and *Ae. albopictus* reaches the highest recall of 99%. The results varied across different species due to the imbalanced nature of each dataset. For the ABUZZ dataset, as shown in Table 1, our model achieved 100% precision, recall, and F1-score for four species, namely *Ae. albopictus*, *Ae. albopicyus*, *Cu. pipiens*, and *Cu. quinquefasciatus*. Two confusion matrices are shown in Fig. 2 with predicted and ground-truth values on horizontal and vertical axes. As shown in the figure, diagonal numbers are correctly classified values and other numbers represent misclassified information. For example, there were 4983 true positive classifications for 5058 *Ae. albopictus* samples on WINGBEATS dataset. Overall, within total 69,893 samples in WINGBEATS, only 7036 cases are misclassified. For the ABUZZ dataset, most of the validation data were correctly classified by our model with only 2 misclassifications over 181 validation data. Overall, it has been found that our model performed better for the ABUZZ dataset compared to the WINGBEATS dataset.

**Figure 3:**
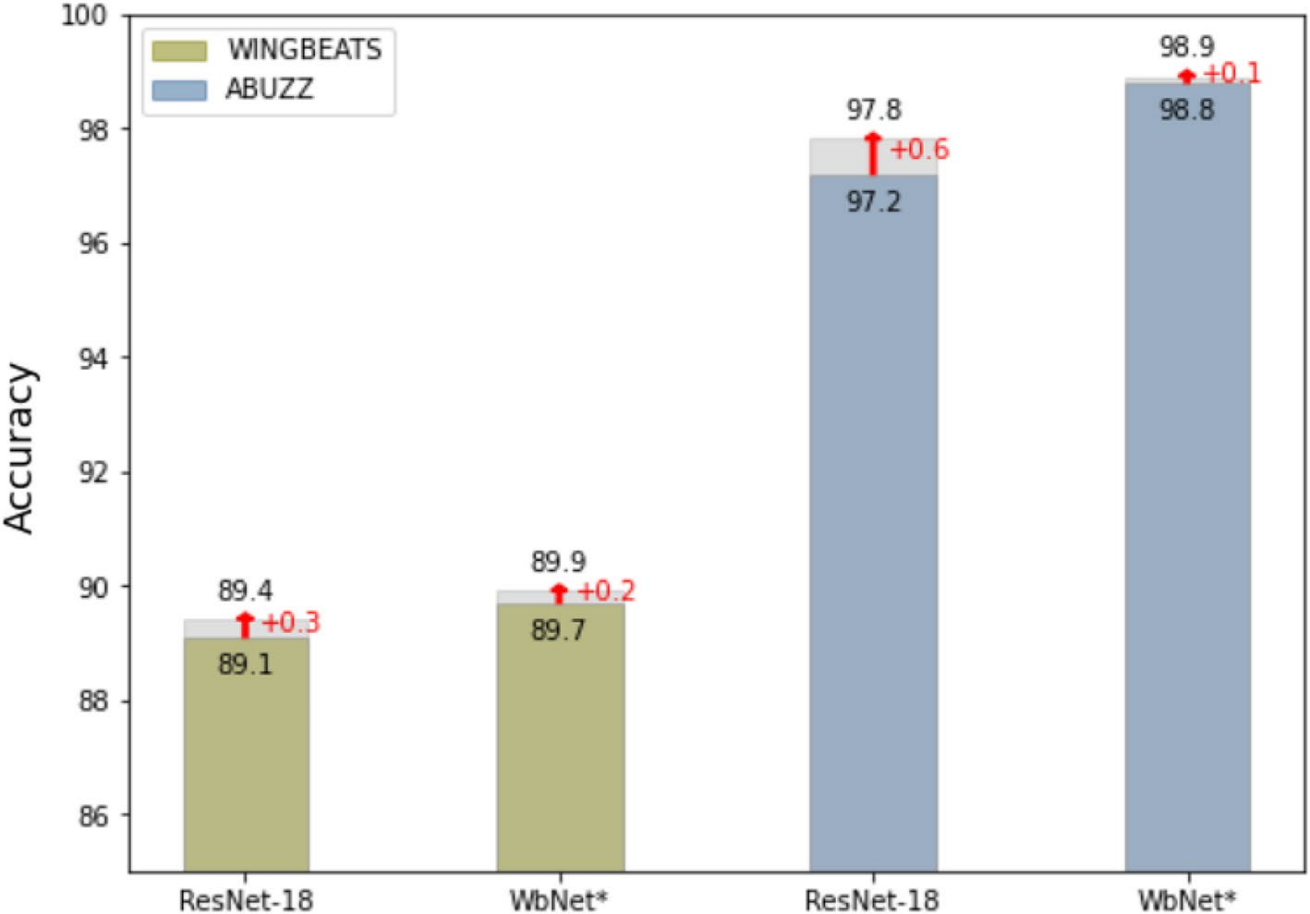
Data augmentation on ResNet-18 and *WbNet* model, percentage increased shown in red.

Lastly, to improve model performance, we implemented a data augmentation technique by applying masks on both time domain and frequency dimensions on Mel-spectrograms to prevent overfitting, and make our model more stable and robust. We executed the augmented and original Mel-spectrogram data within the basic ResNet-18 and our *WbNet* models to test the impact and stability of out data augmentation method, where the result is shown in Fig. 3. We found that, the accuracy of the ResNet-18 model increased by 0.3% on the WINGBEATS dataset. The performance of our *WbNet* model was further improved by 0.2% and 0.1% for WINGBEATS and ABUZZ.

## Discussion

Our project aims to identify different species of mosquitoes by their unique wing beating sounds. We pre-processed the wing beating sounds from two different datasets, tested numerous 1D and 2D ML models, and finally proposed our modified model, namely *WbNet*. The *WbNet* outperforms other existing models on wing beating sounds with the accuracies of 89.9% and 98.9% for respective datasets. A data augmentation method showed a slight increase (0.1–0.2%) in overall performances.

Our first experiment found more features in converted spectrograms than the raw audio sounds. Raw audios are 1D signals, which have memory recession during model progression and some of the spatial dependencies get lost during training. While raw audio contains only one-dimensional time-domain signal, the spectrogram covers both the time domain and frequency distribution information^29^. As a consequence, the subsequent experiment with different ML models with 1D (raw audio) and 2D (spectrogram) inputs showed better accuracies for processed spectrograms. Our finding is similar to Fanioudakis et al^27^, where spectrograms inputs outperformed raw audios.

While comparing different 2D-input based models, namely 2-layer-CNN^26^, ResNet-18^38^, ResNet-34^38^, ResNet-50^38^ and DenseNet-121^39^, ResNet models performed best among the model. The CNN-based models preferentially gather local information to extract features. As the sound wave is a continuous period, some spatial dependencies in terms of global information needed to be considered to enhance the overall accuracies. Subsequently, we build our model on ResNet-18, which performed better than other CNN-based models. A possible explanation is—our model combines the advantages of both state-of-art CNN model and a self-attention mechanism. Our model considered both local and long-term features dependencies problems, which assisted the model to achieve more accurate classification results. Our model employed a self-attention mechanism through the allocation of weight parameters^40^, which is capable to capture the global information and works better for feature extractions. Traditional attention mechanisms calculate attention scores by hidden states between source end and target end and assign different weights on different parts of input to extract the more important information. The self-attention mechanism put more weights on informative parts, which resulted in detailed attention rather than the whole input data^41^. It captures the dependencies within the source or target end itself, which solved the problem of ignoring dependencies within the source end in the traditional attention mechanism and kept other advantages from the traditional attention mechanism. Besides we applied a data augmentation technique into the model, which alleviated the overfitting problem and extended the training time, ensuring better accuracy. However, a DenseNet-121-based model^27^ resulted better (96%) accuracy than our model. While re-implementing their model with our processed spectrograms, the overall accuracy of that model drop to 89.2%, as shown in Fig. 1. The possible reason can be due to different parameter settings during spectrogram conversion or hyperparameters during training.

Besides, the learning rate setting was a crucial and challenging part of our model as the rate controls the speed of a neural network model to learn a problem in each step^42^. The discrepancies in learning values resulted in an unstable training process or sometimes lengthy training period^42^. A learning rate schedule is used to change the learning rate throughout the training process according to a pre-defined schedule. The reduction trend of the cosine learning rate was relatively slow at the beginning, decreasing faster in the middle, and decreasing slowly again until close to zero. As cosine learning rate schedule provides smooth and stable training results, we used the schedule to train our WbNet model.

Further, to increase model performance, a data augmentation method—SpecAugment^43^ has been added into the model to overcome the overfitting problem^44^. In our *WbNet* model, SpecAugment modifies the Mel-spectrogram by masking both frequency and time domain channels and preventing over-fitting by deliberately giving some corrupted data—which increased the robustness of the network for mosquitoes’ wing-beating sound recognition. Data augmentation methods are widely used in mosquito classification models. A CNN-based model on mosquito images got a 23% increase in performance with data augmentation functions of vertical and horizontal flip, random rotation, and noise^13^. Another model got a 13.1% boost^45^ with data augmentation on the mosquito image data. Even though our model for spectrogram gets only 0.1–0.2% increase in overall performances, it showed some potential to utilize similar data augmentation techniques in sound-based classification tasks, to improve their performances.

Overall, raw audios based models provided better results for WINGBEATS than ABUZZ. WINGBEATS audios were short, 0.65 s in length, and captured with a sophisticated audio device, whereas ABUZZ data is longer in length, varies up to 5 min but captured on normal mobile devices within noisy environment. For 1D models, longer audios with noises might effect the feature extraction part of the model, causing degradation in model performance. Interestingly, for spectrogram-based models, we saw the opposite scenario of having better validation results for ABUZZ than the WINGBEATS. Due to the longer length, each ABUZZ audio was split into several segments where each segment was 10-s long. Thus, more data were generated for the ABUZZ dataset, and the classifier block learn more feature information than the WINGBEATS. Since WINGBEATS had lower features than ABUZZ, the deeper neural network models become prone to model overfitting problems, which might resulted in demotion of overall performance.

The current model will be beneficial for ongoing mosquito eradication programs to identify mosquito species prevalence in a target area and propose species-specific measures. As male mosquitoes have a higher wing-beating rate than female mosquitoes^46^, our model can be widely used to classify females mosquitoes, which are solely responsible for transmitting pathogens. Besides mosquitoes, the current model will serve as a baseline model to classify other insect-pest species, based on their unique sound features, e.g.—wing-beating, movement, feeding sounds, etc, and can be adopted for other audio-based classification tasks^47^. Lastly, the current study is based on audios, and we can only detect one species at one time. There is a scarcity of multi-species and gender-specific wing-beating datasets available online. Due to the lack of such a comprehensive dataset, we couldn’t implement and evaluate our model performance on those audios. Future directives of the present study can be constructing a comprehensive wing-beating dataset for multi-species and gender-based classification tasks.

## Methods

### Dataset

Current study utilized two publicly available datasets, namely “WINGBEATS”^24^ and “ABUZZ”^25^. The “WINGBEATS” dataset contains raw audio sounds of six mosquito species of three different genera, namely—*Ae. aegypti, Ae. albopictus, An. arabiensis, An. gambiae, Cu. pipiens, and Cu. quinquefasciatus*. All data were collected individually from six different insectary boxes at the premises of Biogents, Regensburg, Germany, and recorded by large aperture optoelectronic devices^24^. Each audio sound was 0.65 s in length, with a sample rate of 8000 Hz. The number of sound files ranging from 19,297 files for *An. arabiensis* to 85,553 files for *Ae. aegypti*. The second dataset, namely “ABUZZ” was collected from Mukundarajan et al.^25^. The dataset contains wing-beating sounds of twenty mosquito species, spreading over four different genera. However, we only selected the six above-mentioned mosquito species to proceed further. Compared to the previous dataset, the number of sound files was low (8 files for *Cu. pipiens* to 66 files for *An. gambiae*) and all sounds contain a relatively large amount of noises due to publicly sourced, mobile phone recorded audios. The length of most audio sounds varies up to 5 min, with sample rates of 8000 Hz and 44,100 Hz.

### Data pre-processing

From raw audio waves, we first inspected the amplitudes of the data waveform and detected silence or small noises which drastically affect the model performances due to the low magnitude. Hence, for noises with no overlapping part with the mosquito wing-beating sounds, we manually removed the segment using *Audacity*. For both datasets, we adopted unique filtering methods to ensure a uniform dataset for downstream tasks. Further, we padded the data using a ‘reflection padding’ method, where padding the data by reflecting it over the boarding axis. The reflection padding was used to reflect an audio wave into both left and right sides of an original audio wave, where the original audio wave worked as a mirror. It is useful for smooth transition of any audio waves. For the ABUZZ dataset, the number of audio samples was small but lengthy. We divided each audio of ABUZZ into multiple audio segments, to increase the sample number.

Further, we extracted Mel-spectrograms from the waveform audios and then fed Mel-spectrograms as input data into our network. Since an audio signal is a mixture of several frequency waveforms, we used Fast Fourier Transform (FFT) algorithm to transform the audios into individual frequencies. To derive the Mel-spectrogram, we converted the FFT into inter-connected audio segments and then stacked all FFT outputs together to address different frequencies of audio over the time domain. Later, to get the spectrogram, we log-transformed the frequencies and converted the unit of amplitude to decibels. Finally, we applied mel-scale through frequency to obtain Mel-spectrograms. All procedures were done using librosa^48^ library in python. Hence, Mel-spectrograms from audios with their corresponding class labels were stored as our input data and labels of the deep neural network.

**Figure 4:**
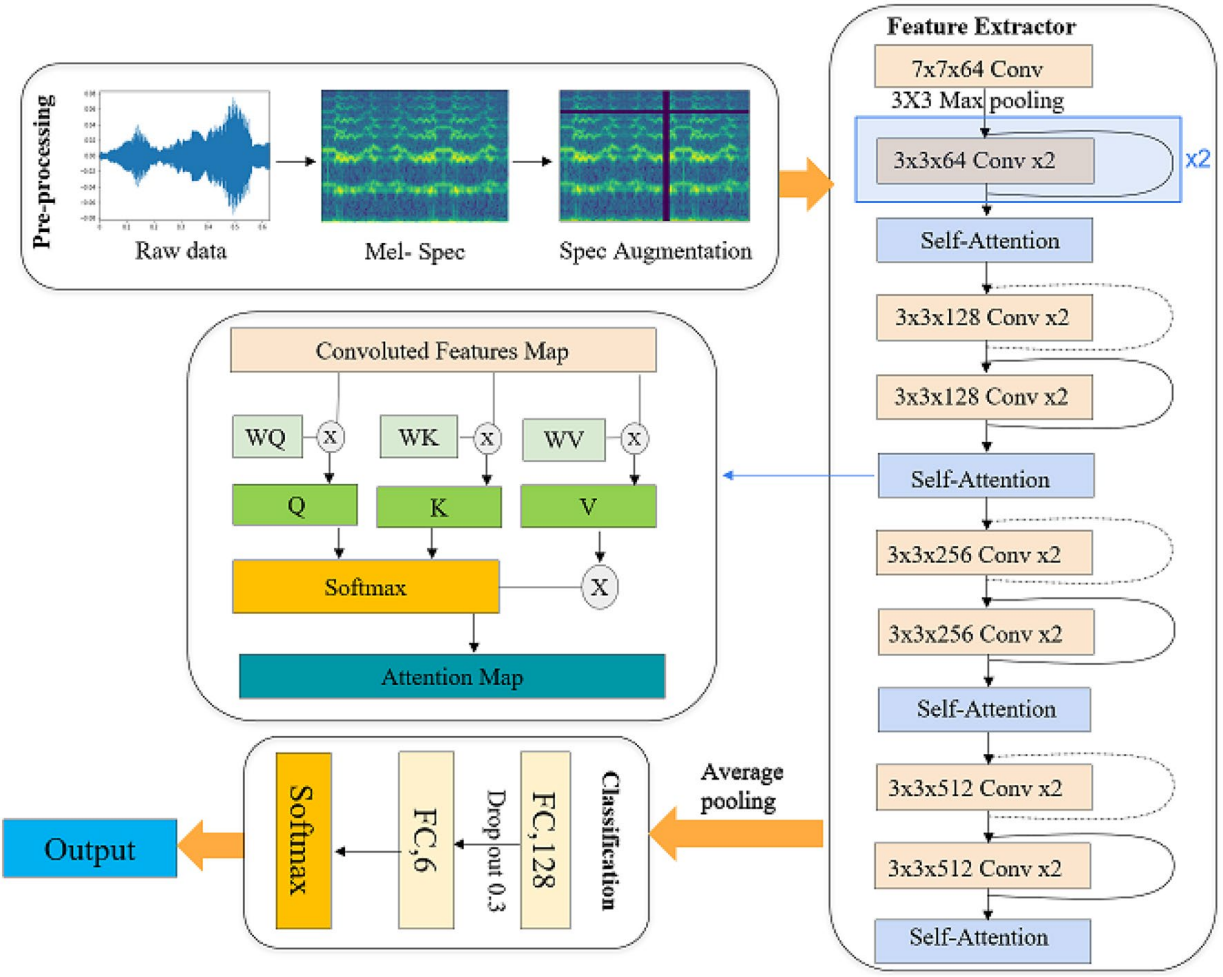
The architecture of our *WbNet* model.

Data augmentation is considered to increase the input audio signals for each species of mosquitoes by generating similar data without collecting more audio signals from scratch. The process works as a regulariser and reduces the chance of over-fitting when training the WbNet. For the data augmentation, we implemented a simplified version of SpecAugment^43^ directly on the processed spectrograms. We modified our spectrograms only by masking data features both on time and on frequency domains. We assumed the time length of an audio was $\tau$, we chose a random number from 0 to $\tau$ as the starting point of a mask, and then select a random number *t* as the masking range, so that all features between $t_{0}$ and $t_{0}+t$ were masked. As shown in the SpecAugment part in Pre-processing at Fig. 4, the horizontal axis was the time domain, where the vertical black rectangle was the mask on the time domain. For frequency masking, we assumed that $\upsilon$ was the frequency channels, and we randomly selected a number *f*0 from 0 to $\upsilon$ as the starting point of the frequency masking and then chose a random number *f* as the range of the masking space. As a result, all features between the *f*0 and $f_{0}+f$ were masked. The masking value of 10 was selected randomly, for making the training results more stable. The overall formula is shown below.

$$\begin{aligned} SpecAugment = {\left\{ \begin{array}{ll} \text {Time Masking:} &{} [t_{0},t_{0}+t],\;\; \text {where}\; t_{0}> 0,\; \text {and} \;t{0}+t< \tau \\ \text {Frequency Masking:} &{} [f_{0},f_{0}+f],\;\; \text {where}\; f_{0} > 0,\; \text {and} \; f{0}+f < \upsilon \end{array}\right. } \end{aligned}$$

### Implementation of 1D and 2D models

We implemented different ML models on mosquito wing-beating sounds from both datasets. DenseNetMLP and 1D-CNN were re-implemented from previous studies^27^ on raw audio sounds, as they accept only 1D inputs. Further, we directly implemented our pre-processed Mel-spectrograms on different ResNet models, such as ResNet-18^38^, ResNet-34^38^, ResNet-50^38^, along with two existing models on mosquito wing-beating sounds (2-layer-CNN and DenseNet-121^27^). The transfer Learning method was applied to ResNet models to speed up training. While 2-layer-CNN^26^ model was built upon twenty-mosquito species, to be consistent across all analyses, we limit the inputs for six species of mosquitoes only.

### Architecture of WbNet

Our model contains a feature extractor and a classification block to classify pre-processed spectrograms. The feature extractor was implemented in a combination with the ResNet-18 network and Self-Attention mechanism, which gathered the leverage of both Residual Networks and Attention. Residual Networks^38^ were adopted to solve the degradation problem, whereas the self-attention mechanism^41^ focused on the spatial dependencies. In general, an attention mechanism focuses on the all relevant features of an input data and the self-attention mechanism is a part of the WbNet architecture that focuses on different features of the winbeating audios in order to compute a better representation of the mosquito. In our model, a self-attention mechanism^49^ was stacked after every two residual blocks to extract more pivotal information and improve model accuracies, by minimizing computational and storage requirements.

In our model, ResNet-18 had been used as a basis. As shown in Fig. 4, in the feature extractor part, every orange block with a curve was a residual block. Residual blocks are important component of ResNet, where F and *x* denote block and input. We fed x into F to get the output F(x), and we applied a shortcut connection from input x to output F(x). A shortcut connection can skip some layers to obtain the final result of the element-wise addition of F(x) and x. Generally there are two shortcut connections in ResNet-18, called convolution shortcut connections (dotted curve) and identity shortcut connections (solid curve), as shown in the *Feature Extractor* block at Fig. 4. Dotted curve was used when output dimensions changed between two sub-blocks in the *Feature Extractor* block, otherwise we used a solid curve. As the dimension was changed for the dot curve, we expanded the input dimension to make them uniform with the output dimension before applying element-wise addition. In each sub-block, we had two convolutional layers with batch normalizations, where the ReLU activation function was used between the layers.

The self-attention mechanism^41^ helps our model to capture more important information through the allocation of weight parameters^40^. Within the self-attention mechanism (Fig. 4), three queries were gained from the dot products between weight matrices ($W_{Q}, W_{K}$, and $W_{V}$) and input of convoluted features map *X*, where Q, K, and V denotes queries, keys, and values. The overall equation is illustrated in Eq. (2). Briefly, we first calculated the attention score by applying dot product between Q and K and then divided by $\sqrt{d_{k}}$. $\sqrt{d_{k}}$ was a scale that prevented the result from the dot product of Q and K being too large. Later, softmax was applied to normalize the score into probabilities to check significant features. We applied the dot product again between the probabilities and the V matrix to get the final score. The main idea for this step was to reduce unimportant features by keeping the attention of important features. In the end, we accumulated weights to produce the output of the self-attention layer.

2 $$\begin{aligned} Attention (Q,K,V) = softmax \left( \frac{QK^{T}}{\sqrt{d_{k}}}\right) V \end{aligned}$$

For the classification block, we applied average pooling from feature extractor outputs to feed the features into a fully connected layer with 128 dimensions. We extracted the features into a 6-dimension fully connected layer applying the dropout technique^50^. The 6-dimension layer was considered due to six mosquito species. A dropout of 30% of units was used to prevent overfitting, which drops some neuron units randomly while training the network. Since the output of the fully connected layer was not normalized, a softmax activation function was introduced into our classifier to obtain the final classification probabilities for each species (Eq. 3). This function can convert a set of numbers into probabilities, which helped to represent a probability distribution over a set of discrete variables^1^. The equation of softmax is shown in Eq. (3), where *z* is the input vector of softmax; in our case, *z* is a vector of the output of a 6-dimension fully connected layer. Also, *i* is the $i_{th}$ element of input *z*; $z_{i}$ is the value of the $i_{th}$ elements, and K is the total element of vector *z*. Hence, we applied the softmax activation function on top of the 6-dimension fully-connected layer.

3 $$\begin{aligned} \sigma (\mathbf {z})_{i} = \frac{e^{z_{i}}}{\sum ^{K}_{j=1}e^{z_{j}}} \end{aligned}$$

We also applied the cosine learning rate schedule^51^, to train our architecture. The mathematical form of the cosine schedule is shown in Eq. (4). In this equation, $\eta _{t}$ is the learning rate in batch t, where T is the batches in total and $\eta$ is the initial learning rate. In the cosine schedule, we scale the range of the learning rate values from zero to $\pi$.

4 $$\begin{aligned} \eta _{t} = \frac{1}{2}\left( 1+cos\left( \frac{t\pi }{T}\right) \right) {\eta } \end{aligned}$$

### Experimental setup and performance evaluation

All the models were implemented on NVIDIA GeForce RTX 2080ti GPU of 11 GB of memory, using python3.7 with supported libraries of Pytorch, librosa, pandas, and numpy. The datasets were split by 80% for training and 20% for validation. For WINGBEATS and ABUZZ, we trained for 100 and 150 epochs. The number of epochs was chosen by running the model with multiple different number of epochs, to avoid overfitting and underfitting problems. Besides, we used adam optimizer, ReLU activation function, and cross-entropy loss function, to evaluate model performances. In our experiments, adam optimizer showed better performance than other optimizers such as SGD, while cross-entropy loss is proper to be used as a measurement in a classification model. We reported model performances using different evaluation matrices as shown in equations from 5 to 8. The evaluation matrices were calculated from confusion matrix. The confusion matrix is formed with True Positive or TP (both predicted values and actual values are positive), True Negative or TN (both predicted value and actual values are negative), False Positive or FP (predicted values are positive but actual values are negative), and False Negative or FN (predicted values are negative but actual values are positive) values. Accuracy (error between predicted and actual values), precision (dispersion of predicted values), and F1-score (harmonic mean of precision and recall) provide model performances at nominal values.

5 $$\begin{aligned} Accuracy=  \frac{TN+TP}{TN+FP+TP+FN} \end{aligned}$$

6 $$\begin{aligned} Precision=  \frac{TP}{TP+FP} \end{aligned}$$

7 $$\begin{aligned} Recall=  \frac{TP}{TP+FN} \end{aligned}$$

8 $$\begin{aligned} F1{\text {-}}score=  2\times \frac{Precision\times Recall}{Precision + Recall} \end{aligned}$$

## Data Availability

The datasets analysed during the current study are available in Kaggle https://www.kaggle.com/datasets/potamitis/wingbeats and Dryad data repositories https://datadryad.org/stash/dataset/doi:10.5061/dryad.98d7s. The codes, used in the current study, are available in github page https://github.com/xutong30/WbNet-ResNet-Attention.
